# Catalytic Combustion of Methane over Pd-Modified La-Ce-Zr-Al Catalyst

**DOI:** 10.3390/ma18102319

**Published:** 2025-05-16

**Authors:** Katerina Tumbalova, Zlatina Zlatanova, Ralitsa Velinova, Maria Shipochka, Pavel Markov, Daniela Kovacheva, Ivanka Spassova, Silviya Todorova, Georgi Ivanov, Diana Nihtianova, Anton Naydenov

**Affiliations:** 1 Institute of General and Inorganic Chemistry, Bulgarian Academy of Sciences, Acad. G. Bonchev Str., bl. 11, 1113 Sofia, Bulgaria; katerinatumbalova@mail.bg (K.T.); shipochka@svr.igic.bas.bg (M.S.); pvlmarkov@svr.igic.bas.bg (P.M.); didka@svr.igic.bas.bg (D.K.); ispasova@svr.igic.bas.bg (I.S.); geoivanov@yahoo.com (G.I.); naydenov@svr.igic.bas.bg (A.N.); 2 Department of Chemistry and Pharmacy, University of Sofia “St. Kliment Ohridski”, 1 James Bourchier Str., 1164 Sofia, Bulgaria; nhzz@chem.uni-sofia.bg; 3 Institute of Catalysis, Bulgarian Academy of Sciences, Acad. G. Bonchev Str., bl. 11, 1113 Sofia, Bulgaria; todorova@ic.bas.bg; 4 Institute of Mineralogy and Crystallography, Bulgarian Academy of Sciences, Acad. G. Bonchev Str., bl. 107, 1113 Sofia, Bulgaria; diana.nihtianova@gmail.com

**Keywords:** Pd catalyst, medium-entropy oxides, La-Ce-Zr-Al oxide support, methane combustion, catalyst regeneration

## Abstract

The present study aims to investigate a Pd catalyst on a complex multi-oxide medium-entropy support interlayer La_2_O_3_-CeO_2_-ZrO_2_-Al_2_O_3_ and its possible use as catalysts for methane abatement applications. The low-temperature N_2_-adsorption, XRD, TEM, XPS, TPD, and TPR techniques were used to characterize the catalyst. The palladium deposition on the supports leads to the formation of PdO. After the catalytic tests, the metal-Pd phase was observed. The complete oxidation of methane on Pd/La-Ce-Zr-Al catalyst takes place at temperatures above 250 °C, and in the presence of water vapor, the reaction temperature increases to about 70 °C. The careful choice of constituent oxides provides a balance between structural stability and flexibility. The alumina and lanthanum oxide ensure the high specific surface area, while the simultaneous presence of zirconia and ceria leads to the formation of a mixed-oxide phase able to interact with palladium ions by incorporating and de-incorporating them at different conditions. The mechanism of Mars–van Kerevelen was considered as the most probable for the reaction of complete methane oxidation. The possibility of the practical application of Pd-modified La-Ce-Zr-Al catalyst is evaluated. The use of a mix of multiple rare and abundant oxides makes the proposed catalyst a cost-effective alternative.

## 1. Introduction

Pollution of the atmosphere is a global problem due to the release of harmful substances in the air, which originate from both natural and anthropogenic sources [1,2,3]. Air pollutants include fine particulate matter (PM), SO_2_, H_2_S, NH_3_, CO, CO_2_, NO_x_, volatile organic compounds (VOCs), etc. [4,5]. Their main sources are chemical and petrochemical enterprises, transport, oil and natural gas processing, energy production, machine industry, printing, food production, and many others [6].

Methane, the primary component of natural gas, is a powerful greenhouse gas that significantly contributes to global warming. It mainly originates from agriculture, coal and natural gas burning, and waste decomposition [7]. Reducing methane emissions can be achieved through different techniques such as thermal and catalytic oxidation. High-efficiency combustion systems optimize methane use, minimizing waste and pollution [8,9,10,11]. Thermal incineration, while effective, produces hazardous byproducts [12,13], whereas catalytic oxidation reduces energy requirements and improves efficiency by using catalysts such as platinum, palladium, and rhodium to oxidize methane at lower temperatures, resulting in fewer toxic emissions [14].

Palladium exhibits greater activity than platinum in methane oxidation and is more resistant to thermal sintering but is vulnerable to catalytic poisons, such as lead or sulfur [15]. Despite this, most catalysts for the methane combustion are palladium-based and sometimes combined with metals or metal oxides. A widely used catalyst, Pd/γ-Al_2_O_3_, offers high surface area but has some disadvantages such as high cost and low thermal stability [16,17,18]. It is known that the PdO/Al_2_O_3_ catalyst is not stable at high temperatures due to PdO reduction and surface area loss, which necessitates the exploration of mixed oxide catalysts [19,20]. La_2_O_3_ is commonly added to enhance PdO stability and preserve γ-Al_2_O_3_’s surface area, improving the catalyst’s durability [21]. Complex catalysts are synthesized by the co-impregnation of Pd and La salts, often with a third element, to obtain a three-element system on γ-Al_2_O_3_ or ceramic supports. These mixed catalysts enhanced methane oxidation at high temperatures. The addition of La_2_O_3_ and BaO stabilizes the alumina surface, while CeO_2_ and NiO prevent PdO reduction [22]. On the other hand, ceria enhances oxygen storage by increasing surface oxygen mobility, and La_2_O_3_ addition reduces the CeO_2_ particle size, prevents sintering, and improves reducibility. In general, Ce-La-based compounds offer good catalytic activity due to their oxygen storage and surface basicity [23]. Reducible oxides like CeO_2_ and TiO_2_ influence catalyst reduction behavior, affecting Pd’s oxidation state and catalytic activity in the Pd/Al_2_O_3_ system [24]. Zirconia prevents sintering, making Ce-Zr mixed oxides appropriate additives for noble metal catalysts on alumina [18,25]. ZrO_2_ enhances CeO_2_’s thermal stability and oxygen storage capacity (OSC), improving oxygen transport and redox cycling [26,27]. Methane oxidation catalysts lose activity due to SO_2_ poisoning, nanoparticle sintering, and water formation. Proper support selection reduces agglomeration, and use of the clean fuel limits SO_2_ poisoning, but water unavoidably forms hydroxyl groups on PdO surfaces [28,29]. An innovative approach is to combine the advantages of the above-mentioned individual oxides within a complex support system to ensure the maximal effectiveness of the palladium catalyst. By carefully designing a medium-entropy oxide support, the catalytic performance could be optimized for specific applications, providing a balance between activity, durability, and cost.

Various methods exist for preparing palladium catalysts, with the sol-gel approach standing out for its ability to produce high-surface-area, porous, and stable solids. This technique enables tuning of the final material’s properties, incorporates active components during gelation, and ensures strong metal-support interaction for enhanced stability [30]. Incorporating the active component involves mixing precursors, polymerizing to form a gel, and using high-temperature treatment to remove volatiles [31]. Impregnation involves soaking the porous support (e.g., SiO_2_, Al_2_O_3_) in an active ingredient solution, followed by drying and calcination [32]. Another approach, co-precipitation, simultaneously precipitates metal cations, forming a mixed hydroxide or oxide, which is then dried and calcined [33].

The choice of the high surface-area support is crucial for catalytic systems, requiring stability and mechanical resistance [34]. Ceramic supports like cordierite [35] and mullite [36] possess high thermal resistance, while zeolites [37] and metal supports (for example, stainless steel) [38] are used in extreme conditions due to their superior thermal conductivity [39]. Ceramics provide a balance of stability and cost efficiency [40], while an alumina interlayer enhances noble metal dispersion [36].

This paper aims to synthesize a Pd catalyst on a complex multi-oxide medium-entropy support interlayer La_2_O_3_-CeO_2_-ZrO_2_-Al_2_O_3_ and to study its catalytic behavior, thermal stability, and the effect of water on its activity regarding its potential application in monolithic catalysts for methane emission reduction.

## 2. Materials and Methods

### 2.1. Catalysts Preparation

The support with the composition La_2_O_3_-CeO_2_-ZrO_2_-Al_2_O_3_ was synthesized by the sol-gel method according to a procedure described in the literature [41]. For this purpose, an appropriate amount of Al[OCH(CH_3_)_2_]_3_ (98%, Sigma–Aldrich, St. Louis, MO, USA) was dissolved in water, and then corresponding amounts of 1 M HNO_3_, Ce(NO_3_)_3_·6H_2_O (99%, Sigma–Aldrich, St. Louis, MO, USA), La(NO_3_)_6_·6H_2_O (99.9%, Sigma–Aldrich, St. Louis, MO, USA), and Zr(NO_3_)_2_·H_2_O (99.9%, Sigma–Aldrich, St. Louis, MO, USA) were added to obtain a gel with a composition of 80 wt.% Al_2_O_3_, 8 wt.% CeO_2_, 4 wt.% La_2_O_3_ and 8 wt.% ZrO_2_. The mixture was stirred at 100 °C for 72 h. The prepared viscous gel was dried at room temperature and calcined in a muffle furnace at 500 °C for 4 h in air. The synthesized material was tableted on a Specac 25.011 press, after which the tablets were crushed and sieved to obtain a fraction with a size between 0.3 mm–0.6 mm. Catalyst containing 2 wt.% Pd was obtained by impregnation with a solution containing Pd(CH_3_COO)_2_ (40%, Sigma–Aldrich, St. Louis, MO, USA) dissolved in acetone. The sample was shaken until the complete evaporation of the acetone and calcined in a muffle furnace at a temperature of 450 °C for 2 h. This sample was denoted as fresh Pd/La-Ce-Zr-Al.

A second type of core-shell catalyst was prepared to evaluate the possibilities for practical use. It was obtained by gel deposition with the composition La_2_O_3_-CeO_2_-ZrO_2_-Al_2_O_3_ on cylindrical mullite-based ceramic extrudates (4 mm diameter and length from 8 to 10 mm) and further heated for 15 min at 120 °C and calcined at 475 °C for 3.5 h. The catalyst containing 0.5 wt.% Pd was obtained by impregnation with a solution containing Pd(CH_3_COO)_2_ in acetone, followed by thermal treatment at 450 °C for 2 h. This sample was denoted as Pd/La-Ce-Zr-Alc.

### 2.2. Characterization Techniques

The BET method [42] was used to measure the specific surface areas of the samples through N_2_ adsorption isotherms at 77 K, utilizing a NOVA 1200e (Quantachrome Instruments, Anton Paar, Boynton Beach, FL, USA). Prior to analysis, the samples were vacuum-degassed at 200 °C for 18 h. Total pore volumes were assessed at a relative pressure close to unity, while the pore size distributions were calculated using the Barrett–Joyner–Halenda (BJH) method [43], based on the desorption branches of the isotherms.

A D8 Advance diffractometer Bruker (Karlsruhe, Germany) with a LynxEye detector and Cu Kα radiation was used to perform X-Ray diffraction (XRD). The diffractograms were recorded within the 5–80-degree 2θ. Using the ICDD-PDF2 (2021) database and Diffracplus EVA, phase identification was carried out. The Topas-4.2 program was used to evaluate the unit cell parameters, mean crystallite size, and amounts of crystalline phases in the samples.

Transmission electron microscopy (TEM) studies were performed on a high-resolution scanning transmission electron microscope HR STEM JEOL JEM 2100 (JEOL Ltd., Tokyo, Japan). The samples were prepared on standard C/Cu grids.

The electronic structure and surface composition of the catalyst were analyzed using X-Ray photoelectron spectroscopy (XPS) with an AXIS Supra spectrometer (Kratos Analytical Ltd., Manchester, UK). Measurements were taken with AlKα radiation (1486.6 eV) and a charge neutralization system. Binding energies were calibrated using the C1s peak at 284.6 eV. The surface composition was determined by analyzing photoelectron peaks (C1s, O1s, La3d, Ce3d, Zr3d, Al2p, and Pd3d), and element concentrations (atomic%) were calculated using ESCApe™ 1.2.0.1325 (Kratos Analytical A Shimadzu, Stretford, UK) software by normalizing the peak areas to sensitivity factors.

A gas analyzer Teledyne Mod. 802 was used to collect oxygen temperature programmed desorption (O_2_-TPD) data. The sample was treated to 450 °C in a 5% O_2_ in N_2_ flow for 6 h. It was cooled to room temperature using the same gas mixture. The N_2_ gas flow was 500 mL/min^−1^, and the heating rate was 10 K/min^−1^. After the O_2_-TPD, temperature-programmed reduction by methane (CH_4_-TPR) tests were carried out with 0.125 vol.% methane in the same gas flow. An online gas analyzer (MultiGas FTIR Gas Analyzer 2030G, MKS Instruments Inc., Andover, MA, USA) was used to perform the gas analysis for the TPR tests. The compositions of the various reaction gas combinations were set up using a Bronkhorst multi-channel, mass-flow controller device.

### 2.3. Catalytic Activity Measurement

A continuous-flow laboratory reactor was used for the catalytic activity tests, which were carried out with the following parameters: a catalyst volume of 0.7 cm^3^ (a mix of 0.5 cm^3^ of catalyst and 0.2 cm^3^ of quartz glass beads) and irregularly shaped particles with an average diameter of 0.45 ± 0.15 mm. The quartz-glass inner reactor has a diameter of 6.0 mm (D_r_/D_p_ ≥ 10). After testing to determine the conditions for minimizing the constraints due to the external mass transfer, the velocity GHSV_STP_ was set to 60,000 h^−1^. The volume of the core–shell catalyst was 1 cm^3^ with GHSV_STP_ = 30,000 h^−1^. A constant reaction temperature (deviation: ±1 °C) was maintained. The inlet concentrations of the gases were varied as follows: methane—0.1 vol.%, oxygen—5 vol.%, and water vapor—2 vol.%. All feed gas mixtures were balanced to 100 vol.% with nitrogen (4.0). Gas analyses were conducted using specialized online gas analyzers for CO/CO_2_/O_2_ (Maihak-Sick Mod. 710S, NDIR) and THC-FID (Thermo FID-TG, SK Elektronik GmbH, Leverkusen, Germany).

The sample denoted as worked Pd/La-Ce-Zr-Al is the sample after catalytic tests in the presence of water vapor.

The sample denoted as “after thermal aging” is a fresh sample that first underwent thermal treatment (170 h in air at 500 °C in the absence of water), followed by the same catalytic tests in the presence of 2 vol.% H_2_O.

## 3. Results and Discussion

### 3.1. Catalytic Tests on Pd/La-Ce-Zr-Al and Pd/La-Ce-Zr-Alc Catalysts

**Figure 1:**
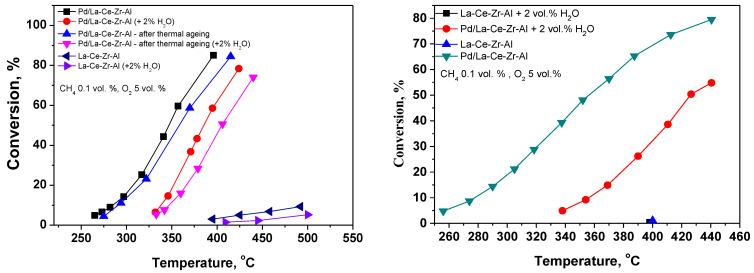
Catalytic activity of the Pd/La-Ce-Zr-Al catalyst and La-Ce-Zr-Al support in the reaction of complete methane oxidation with and without water vapor and after thermal aging (**left**) and tests on the Pd/La-Ce-Zr-Alc catalyst (**right**).

Figure 1(left) presents the results from the tests for the complete oxidation of methane on the Pd/La-Ce-Zr-Al catalyst (fresh, and after thermal aging) with and without water vapor. The term “conversion” is used for representing the degree of methane that is converted to CO_2_ and H_2_O (complete oxidation), i.e., when no byproducts (such as CO, other organic compounds such as aldehydes, acids, etc.) are formed during the reaction.

The complete oxidation of methane takes place at temperatures above 250 °C. The required temperature for the conversion of 50% of methane (T_50_) in the absence of additional water vapor is 346 °C, while in the humid gas feed (2 vol.% water vapor), the T_50_ is 384 °C.

The thermal stability of the samples was evaluated by aging them at 500 °C for 170 h, with and without water vapor. The increase in the T_50_ was applied as a quantitative measure for stability. It was observed that the thermal aging did not significantly influence the catalytic activity with and without water vapor (the increase in T_50_ has a value of 30–35 °C, compared in Figure 1’s black–blue and red–magenta curves).

Figure 1 presents the test results for the complete oxidation of methane with and without water vapor for the Pd/La-Ce-Zr-Alc catalyst. The reaction of the complete oxidation of methane proceeds at temperatures above 250 °C. The T_50_ without added water vapor is 356 °C, while with 2% water vapor, the T_50_ is 426 °C. The support itself has almost no catalytic activity.

The behavior of similar systems was reported earlier [18,44,45]. Regarding the Ce-Zr-Si-Pd and Ce-Zr-Pd catalysts [45], the comparison of the activities shows close values for T_50_. The calculations are performed after the mathematic model recalculation of the conversions from GHSV = 75,000 h^−1^ (literature data, Khan at all. [45]) to GHSV = 60,000 h^−1^ (present study).

### 3.2. Physico-Chemical Characterization of the Catalysts

The textural characteristics of the La-Ce-Zr-Al support and the Pd/La-Ce-Zr-Al catalyst were determined using nitrogen physisorption.

**Figure 2:**
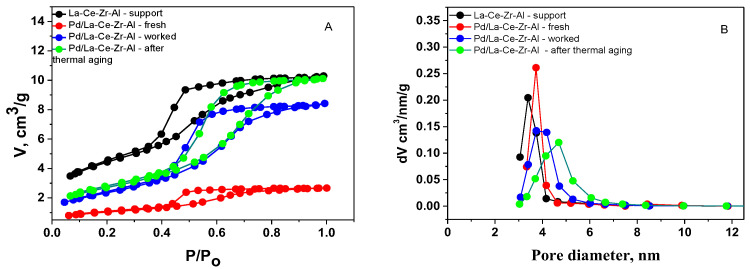
Adsorption/desorption isotherms (**A**) and PSDs (**B**) of the La-Ce-Zr-Al-support, Pd/La-Ce-Zr-Al-fresh, Pd/La-Ce-Zr-Al-worked, and Pd/La-Ce-Zr-Al- after thermal aging catalysts.

Figure 2A presents the adsorption–desorption isotherms, and Figure 2B presents the pore size distributions (PSDs) of the support and catalysts (fresh, after catalysis, and after thermal aging).

The results reveal that all samples, according to the IUPAC classification, show a type-IV isotherm that is typical for mesoporous materials [46]. Mesoporous materials, characterized by pore sizes ranging from 2 to 50 nm, provide unique advantages in enhancing catalytic performance due to their ability to facilitate the transport of reagents and products.

The data in Table 1 show that the volume of the mesopores of the support is about 0.2 cm^3^/g, and the specific surface area is 188 m^2^/g. After palladium deposition, the measured S_BET_ of the catalyst is 158 m^2^/g. Because the total pore volumes of the support, the fresh sample, and the sample after catalytic tests and after thermal aging remain practically unchanged, it can be assumed that the deposited palladium has high dispersity. The pore volume of the overall catalyst (support + active palladium-containing phase) is slightly decreased, which reveals that the palladium phase particles are fine and do not fill or block the pores. Figure 2B shows that the pore-size distribution is monomodal. The pore size distributions of the La-Ce-Zr-Al support, the fresh Pd/La-Ce-Zr-Al, and the worked Pd/La-Ce-Zr-Al catalyst are narrow, indicating the presence of agglomerates of small particles. In the sample after thermal aging and after catalysis for Pd/La-Ce-Zr-Al (Figure 2B), a slight shift of the maximum pore diameter toward higher values is observed due to some particle aggregation, which is also expressed in a decrease in the specific surface area.

**Figure 3:**
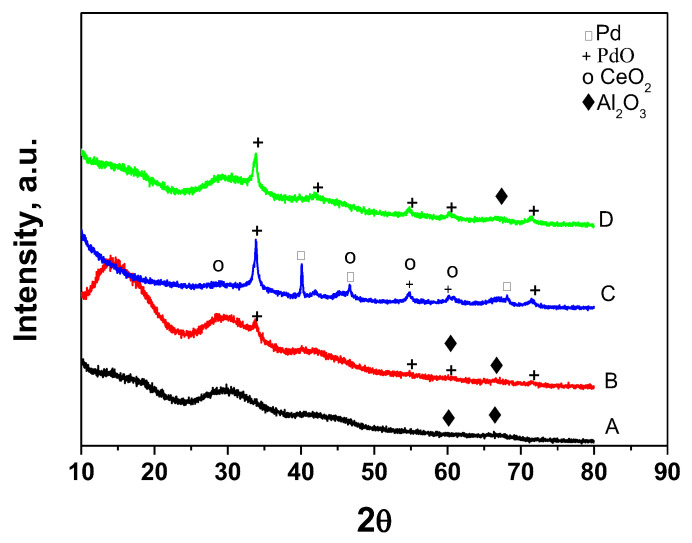
XRD patterns of La-Ce-Zr-Al—support (A), Pd/La-Ce-Zr-Al—fresh (B), Pd/La-Ce-Zr-Al—worked (C), and Pd/La-Ce-Zr-Al—thermal aging (D).

The X-Ray diffraction patterns of the support, fresh, worked, and thermally aged Pd/La-Ce-Zr-Al catalysts can be seen in Figure 3. Table 2 shows the mean crystallite size and phase composition of the Pd/La-Ce-Zr-Al catalyst. The diffraction pattern of the support La-Ce-Zr-Al is typical for amorphous Al_2_O_3_. Thus, the amorphous state of alumina ensures the high specific surface area of the synthesized support. XRD analysis did not reveal the presence of peaks attributed to rare-earth and zirconium-containing phases, indicating that they are in a highly dispersed state on the alumina [47]. When PdO is deposited on the support, peaks typical of this phase are seen in the XRD pattern corresponding to tetragonal PdO (ICDD-PDF 41-1107).

The unit cell parameters (see Table 2) are similar to those reported in [48].

**Figure 4:**
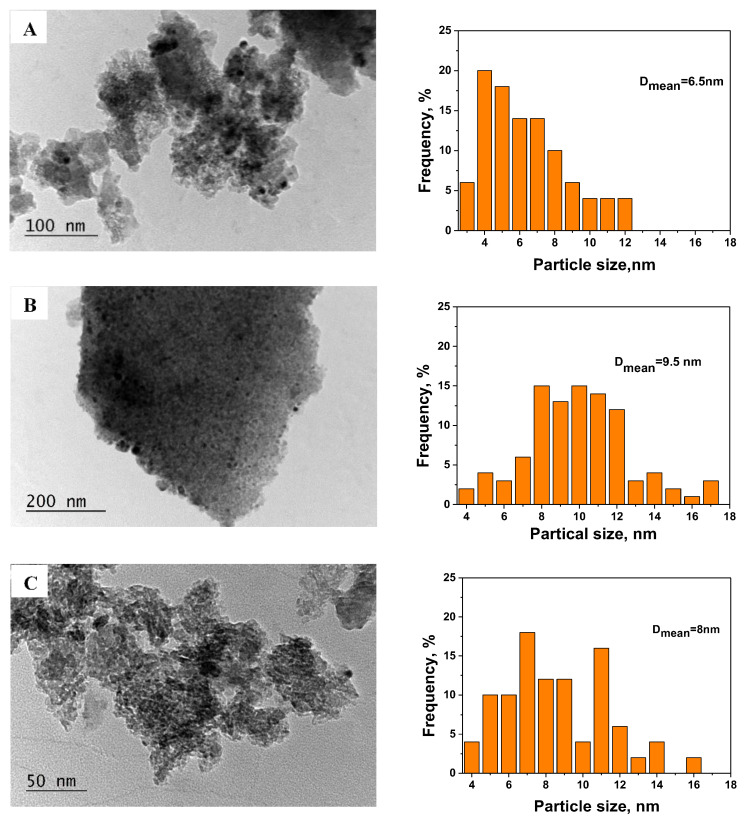
Bright-field images and histograms of particle size distribution for Pd/La-Ce-Zr-Al—fresh (**A**), Pd/La-Ce-Zr-Al—worked (**B**), and Pd/La-Ce-Zr-Al—thermal-aged (**C**).

It should be mentioned that PdO’s diffraction peaks are broad and small, confirming that it is well dispersed on the support. The pattern of the worked sample, in addition to peaks of the γ-Al_2_O_3_ phase, shows weak peaks of cubic-type phase, with a parameter slightly lower for CeO_2,_ indicating that some Zr or Pd ions are incorporated in the CeO_2_ lattice. A new Pd^0^ phase (ICDD-PDF 46-1043) with a high crystallinity appears in the pattern of the sample after the catalytic test, which disappears after 170 h of thermal-aging treatment at 500 °C. The morphology and particle size distribution of the Pd/La-Ce-Zr-Al catalyst were studied by TEM, and the results are displayed in Figure 4.

According to the observations, palladium particles were rather uniformly distributed across the La-Ce-Zr-Al support and appeared as black dots on the catalyst images. Based on 100 randomly chosen nanoparticles, the average particle size in the fresh catalyst was determined as 6.5 nm, which is in concordance with the crystallite sizes from XRD. The average size increases to 9.5 mn after the catalytic test. The average particle size for the sample after thermal aging (8 nm) slightly differs from the fresh and worked samples, showing some peculiarity in its size distribution. Zhou et al. [49] reported that the presence of ceria and zirconia affects the sintering of the PdO_x_ particles due to the enhanced thermal stability of the support.

**Figure 5:**
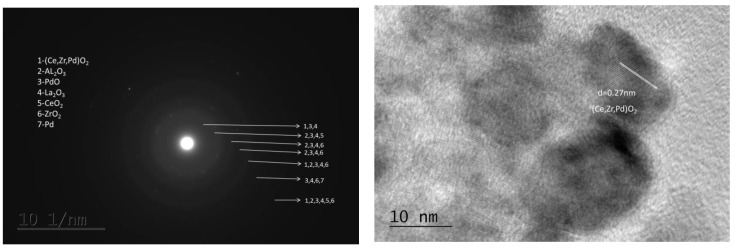
SAED pattern (**left**) and HRTEM (**right**) of the Pd/La-Ce-Zr-Al –worked catalyst.

The selected-area electron diffraction (SAED) pattern of the worked Pd/La-Ce-Zr-Al sample shows the presence of the following (Figure 5 left): (Ce,Zr,Pd)O_2_, γ-Al_2_O_3_, PdO tetragonal, La_2_O_3_, CeO_2_, ZrO_2_, and metallic Pd. Because La_2_O_3_ was not detected in the XRD, it is assumed that it is in a highly dispersive state and contributes to the increase in the specific surface area of the support. Additionally, the presence of the cubic CaF_2_-type phase was found by HRTEM with the probable composition (Ce,Zr,Pd)O_2_ (Figure 5 right). It should be pointed out that Ce-Zr-Pd mixed oxides were also detected by XRD analysis, irrespective of their small amount and high dispersity.

XPS analysis was performed to evaluate the content and the oxidation state of the ions on the surface of the Pd/La-Ce-Zr-Al. Spectra of O, Pd, Ce, La, Zr, and Al were detected on the surface. The surface compositions of the studied catalyst, both fresh and after the catalytic experiments, are shown in Table 3.

**Figure 6:**
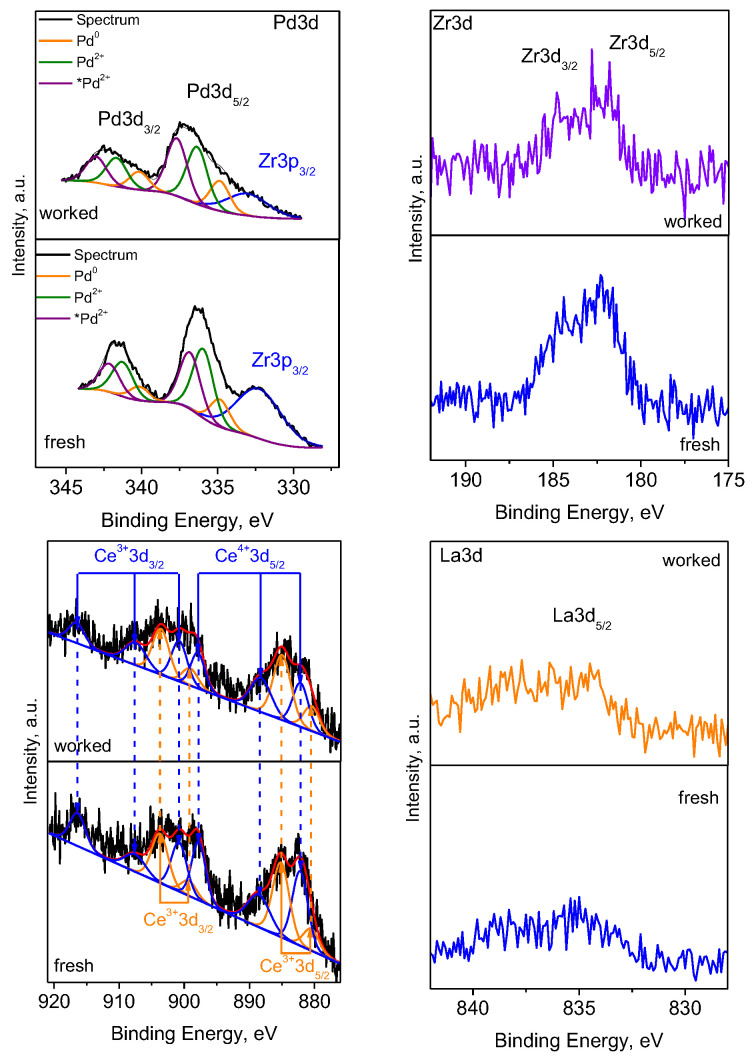
XPS spectra of the Pd 3d, Zr 3d, Ce 3d and La 3d for fresh and worked catalysts.

It should be noted that the palladium content on the surface is greater than the nominal preset content. Hence, it is situated on the surface rather than in the bulk. Regarding the other metals, which are components of the support, it is expected that their surface content is lower than their preset ones. It should be noted that the increased oxygen content demonstrates that the metals on the surface are in oxidized states. The differences in the surface compositions of the fresh and worked catalysts show that the catalyst undergoes redox changes during the catalytic reaction. The XPS spectra of Pd 3d, Ce 3d, Zr 3d, and La 3d are presented in Figure 6.

The binding energies of Pd 3d_5/2_ are registered at 335.1 (±0.2) eV, 336.6 (±0.2), and 337.8 (±0.2) eV for the fresh catalyst and at 334.8 (±0.2), 336.4 (±0.2), and 337.5 (±0.2) for the worked one, respectively. The first two binding energies correspond to metallic palladium Pd^0^ [50] and to Pd^2+^ in PdO [51,52,53]. The third component is interpreted as *Pd^2+^ (Pd^2^^+^, while higher in energy, is not necessarily in a new oxidation state but in a modified electronic environment) in (Ce,Zr,Pd)O_2_, which is also registered in XRD, respectively. A theoretical study [54] predicted that in Pd-doped CeO_2_, the ground-state structure is a square planar, with Pd ions in a d^8^ configuration. This suggests that the Pd^2^^+^ in the Pd-O-Ce solid solution is in a highly ionized state (denoted as *Pd^2+^), which is more highly ionized than those in the standard PdO. A similar finding is reported by Khader et al. [55].

The peak at about 333.3 eV in the Pd3d spectra is ascribed to Zr 3p_3/2_, indicating the presence of ZrO_2_ [56]. This compound is also confirmed by the Zr3d spectra with a peak situated at 182.2 eV [57]. The XPS spectra in the Ce3d region display the Ce 3d_5/2_–Ce 3d_3/2_ peaks, and their curve deconvolution reveals the presence of both oxidation states Ce^3+^ and Ce^4+^ [58]. Due to its low surface concentration, the La3d spectrum presents an ill-resolved La3d_5/2_ peak. The literature data reveal that the splitting between the primary and the satellite determines whether La(OH)_3_ or La_2_O_3_ are available. The splitting of 3.5 eV characterizes La(OH)_3_, while 4.5 eV is reported for La_2_O_3_ [59]. In the present spectrum, this splitting is about 4.1 eV for the fresh catalyst and about 3.6 eV for the worked one, implying that along with La_2_O_3_, partially hydrated La-containing phases are available on both catalysts.

Table 4 provides the percentages of the Pd and Ce in different oxidation states as found on fresh and worked catalysts. The analyses reveal several oxidation states of palladium: Pd^0^, Pd^2+^, and *Pd^2+^ (from (Ce,Zr,Pd)O_2_), respectively. In the worked catalyst, the content of Pd^0^ is enhanced, which is in concordance with the XRD and TEM data. The increase in the *Pd^2+^ content at the expense of Pd^2+^ for the worked sample could be explained by the fact that, during the reaction, palladium interacts intensively with the support, leading to the insertion of Pd ions into the (Ce,Zr)O_2_ lattice. It was also determined that after catalytic tests, the concentration of Ce^3+^ increases due to the redox process.

### 3.3. O_2_–Temperature-Programmed Desorption (TPD) and CH_4_-Temperature-Programmed Reduction (TPR)

**Figure 7:**
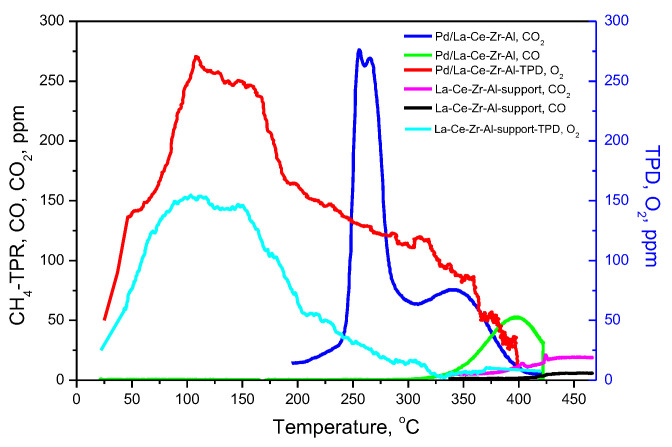
O_2_-TPD and CH_4_/TPR experiments on the Pd/La-Ce-Zr-Al catalyst.

Data for the adsorption and reduction of the catalyst were collected using the O_2_-TPD and CH_4_/TPR studies (Figure 7). The chosen temperature range, restricted to 430–440 °C, is similar to the one used in other catalytic activity experiments [60]. Regarding the support, the O_2_-desorption begins at room temperature and proceeds with a measurable rate up to 330 °C, the most intensive region being observed within the range of 50–200 °C (broad peak with a maximum of 110 °C). The deposition of palladium leads to a significant increase in the amount of the released oxygen, and quantitatively, one can account for a 2.25 times higher amount of desorbed oxygen. Furthermore, the oxygen desorption proceeds intensively up to 400 °C. The O_2_-desorption curve can be deconvoluted into two peaks (at 125 °C and 295 °C), thus revealing two types of surface oxygen species.

CH_4_-TPR spectra show that the complete methane oxidation in the absence of an oxidant in the gas phase proceeds at approximately 220 °C, which is visible from the formation of CO_2_ and produced by the reaction of gas-phase methane and the oxygen from the catalytic surface. A low-temperature peak centered at 265 °C and a broadened high-temperature peak at about 341 °C present the two main peaks of the CO_2_ formation curve. Similar results are obtained by Stasinska using CH_4_/TPR testing [61].

When the reactive oxygen (not precisely distinguished as lattice, surface, or adsorbed oxygen) from the low-temperature peak is depleted, the type of methane oxidation reaction changes from complete to partial, thus resulting in the additional production of CO (beginning at 300 °C). The evolution of oxygen with decreased reactivity can be linked to the second peak of CO_2_ (at 341 °C). It should be noted that some CO_2_ formation occurs at the same temperatures on the support (La-Ce-Zr-Al) sample but with low intensity (>400 °C). Therefore, the existence of two kinds of surface oxygen species may be suggested: (i) highly reactive and mobile species, associated with the Pd-containing active phases, and (ii) lower-activity oxygen from the catalytic support.

### 3.4. Reaction Kinetics of Pd/La-Ce-Zr-Al Catalyst

To obtain data for the elucidation of the reaction mechanism, the experiments were carried out by varying the inlet reaction parameters. The sum of squares between the measured experimental points and the calculated data by any model (RSS) and the squared correlation coefficient (R^2^) were taken as optimization criteria [62,63]. A suggestion of a significant role of the chemisorption of oxygen was made on the basis of the values of the reaction order with respect to oxygen (Table 5—power law kinetics model). Practically, within the range of the oxygen levels, the reaction rate does not depend on the gas-phase oxygen concentration, and the entire reaction proceeds via the oxygen on the catalytic surface. The reaction order toward the water vapor has a negative value (−0.33), revealing a remarkable inhibition effect. To explore the reaction mechanism, two main kinetics equations were selected: Langmuir–Hinshelwood and Mars–van Krevelen (Table 6 and Table 7) [64,65]. The Langmuir–Hinshelwood mechanism [66] involves only reagents in their adsorbed states, while for the Mars–van Krevelen type of mechanism [64], the bulk oxygen from the active phase participates in the reaction, and the main hypothesis is that the rate-limiting step is the reduction of the oxidized catalytic surface. The results from the kinetic calculations in the present case show that the lowest values for the RSS criteria and the highest R^2^ correlation are obtained for the Mars–van Kerevelen model. An additional assumption is that the water is adsorbed on both the oxidized and reduced sites and in the subsequent slow desorption of products (MVK-SDP). Therefore, the Mars–van Krevelen mechanism is more adequate to the experimental results than the alternative Langmuir–Hinshelwood mechanism, in which a competition between the water, oxygen, and methane exists.

After the catalytic reaction, a new crystalline cubic phase of metallic Pd appears, along with the PdO phase. At the same time, the reaction increases the incorporation of Pd into the fluorite-type (Ce,Zr,Pd)O_2_ phase. The thermal aging regenerates the active phase by oxidizing the metal Pd back to PdO. TEM histograms indicate that thermal aging has a negligible effect on Pd particle size. The sample after thermal aging showed almost the same activity as the fresh one, confirming the restoration of the active phase. This restoration is assumed to be influenced by the presence of zirconium ions, which compete with the palladium ions for incorporation in the CeO_2_ lattice. Thus, the proposed support is beneficial for multiple uses, as its regeneration tends to be performed easily. The slight activity decrease after thermal aging can be related to increased PdO particle sizes (as shown by XRD and TEM).

### 3.5. Reactor Modeling

To obtain information about the practical potential of the synthesized material, methane combustion was simulated with a two-dimensional heterogeneous reactor model at semi-adiabatic conditions, taking into account heat losses in the reactor walls. The system of PDEs (partial differential equations) for mass and heat transfer balances was solved in Excel^®^ by the finite differences method [67]. The graphic output was realized by using OriginPro 8.6 (64 bit) software. The visualization of the temperature and concentration profiles was performed by using different colors from blue for the lowest values to red for the highest values. A second-order approximation was used for the numerical solution [67,68,69,70,71,72,73]. The conversion degree at the outlet of the rector was calculated by using the method of mixing-cup average concentration [69]. It consists of multiplying the concentrations of the gas streamlines by the corresponding volumetric flows, summing up all the streamlines, and dividing this sum by the total volumetric flow—a very detailed analysis is given in the literature [67,69].

**Figure 8:**
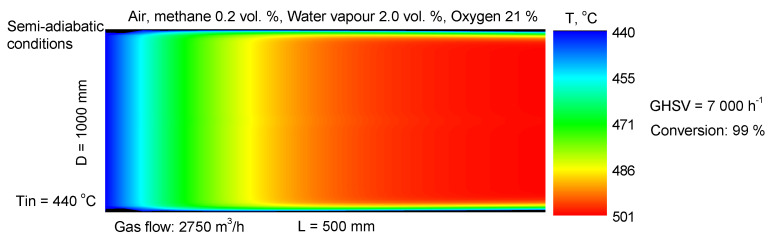
Full-scale reactor model for methane combustion accounting for heat loss at the reactor walls.

The results presented in Figure 8 demonstrate that 99% conversion of methane in a 2750 Nm^3^/h gas mixture (CH_4_: 0.20 vol.%, H_2_O: 2.1 vol.%, 21 vol.% O_2_) is achieved with reactor dimensions D = 1.0 m and L = 0.5 m. Therefore, for efficient neutralization of methane in the presence of water, the reactor must operate in an adiabatic mode at a GHSV of 7000 h^−1^.

The reactor model allows the calculation of the needed length of the reactor if a smaller diameter is required or a larger gas flow has to be treated. For example, if the reactor diameter has to be 0.3 (instead of 0.5 m), then the needed reactor length to achieve 99% conversion is 1.3 m (initially 1.0 m); the corresponding GHSV is 6900 h^−1^. In this case, an increase in bed pressure drop should be expected. If the gas flow increases from 2750 m^3^/h to 3550 m^3^/h, i.e., GHSV changes from 7000 h^−^^1^ to 9000 h^−^^1^, then the needed length of the reactor will be 0.65 m at a fixed diameter of 1.0 m. Alternatively, the reactor diameter should be 1.13 m (at a fixed length of 0.5 m). Therefore, the applied reactor model could be used for the further optimization of the reactor dimensions. A very detailed theoretical hydrodynamic analysis of the reactor conditions has been given by Armenta at all [74].

## 4. Conclusions

A Pd catalyst on the complex, multi-oxide, medium-entropy support interlayer La_2_O_3_-CeO_2_-ZrO_2_-Al_2_O_3_ was successfully prepared. The complete oxidation of methane on Pd/La-Ce-Zr-Al catalyst takes place at temperatures above 250 °C, and in the presence of water vapor, the reaction temperature increases to about 70 °C. It was found that the careful choice of constituent oxides provides a balance between structural stability and flexibility. The alumina and lanthanum oxide ensure the high specific surface area, while the simultaneous presence of zirconia and ceria leads to the formation of a mixed-oxide phase able to interact with palladium ions by incorporating and de-incorporating them at different conditions. The combined effect of the support is that the catalyst is resistant to the sintering of the active phase or degradation under reaction conditions, and its thermal stabilization is slightly influenced by the water vapor. This effect provides the opportunity for facile catalyst regeneration and its multiple uses. It is found that complete methane oxidation proceeds most likely via the Mars–van Kerevelen mechanism. An evaluation of its potential for practical use in the development of monolithic catalysts based on Pd/La-Ce-Zr-Al was made. The use of a mix of multiple rare and abundant oxides makes the proposed catalyst a cost-effective alternative.

## Figures and Tables

**Table 1:** Textural characteristics of the obtained La-Ce-Zr-Al support and the Pd/La-Ce-Zr-Al catalyst.

Sample	S_BET_ m^2^/g	V_t_ cm^3^/g	D_av_ nm
La-Ce-Zr-Al—support	188	0.19	4.1
Pd/La-Ce-Zr-Al—fresh	158	0.18	4.6
Pd/La-Ce-Zr-Al—worked	115	0.17	6.0
Pd/La-Ce-Zr-Al—thermal aged	112	0.18	6.5

**Table 2:** Phase composition with unit cell parameters and crystallite sizes.

Sample	Amorphous	γ-Al_2_O_3_	(Ce, Zr, Pd)O_2_ Cubic Fm-3m	PdO Tetragonal P42/mmn	Pd Cubic Fm-3m
La-Ce-Zr-Al—support	96%	broad diffuse peaks 4%	- <2 nm	-	-
Pd/La-Ce-Zr-Al—fresh	91%	broad diffuse peaks 4%	- <2 nm	a = 3.042(7) Å c = 5.35(3) Å 4% 9nm	-
Pd/La-Ce-Zr-Al—worked	-	86% 3 nm	5.34(3) Å 8% 3 nm	a = 3.03(1) Å c = 5.36(3) Å 4% 18 nm	3.89(1) Å 2% 68 nm
Pd/La-Ce-Zr—thermal aging	96%	broad diffuse peaks 3% 4 nm	- <2 nm	a = 3.044(2) Å c = 5.340(4) Å 2% 19 nm	-

**Table 3:** Surface composition at.%.

Samples	O	Pd	Ce	La	Zr	Al
Nominal preset	40.2	2	6.5	3.4	5.9	42
Pd/La-Ce-Zr-Al fresh	56.5	6.6	1.2	0.5	3.6	31.6
Pd/La-Ce-Zr-Al worked	55.3	7.2	1.4	1.0	3.3	31.8

**Table 4:** XPS results of the oxidation states of Pd3d_5/2_ and Ce3d.

Samples	Pd^0^ (%)	Pd^2+^ (%)	*Pd^2+^ (%)	Ce^4+^ (%)	Ce^3+^ (%)
Pd/La-Ce-Zr-Al fresh	17.1	45.6	37.3	54.3	45.7
Pd/La-Ce-Zr-Al worked	19.7	40.0	40.3	40.0	60.0

**Table 5:** Kinetics parameters based on the power law model.

PWL $r=kC_{voc}^{m}C_{ox}^{n}C_{water}^{p}$							
	E_a_	k_o_	m (CH_4_)	n (O_2_)	p (H_2_O)	RSS	R^2^
Pd/La-Ce-Zr-Al	122.8	8.40 × 10^10^	1.02	0.01	−0.33	15.5	0.999

E_a_, kJ/mol; k_o_, mol.s^−1^m^−3^; k = k_o_.exp(−E_a_/RT); k, mol.s^−1−[1−(m+n+p)]^; RSS—squared sum of residuals; R^2^—squared correlation coefficient.

**Table 6:** Reaction rate expressions and kinetics parameters for the applied LH model.

LH-DS-D: Water Compete with Oxygen and Methane $r=\frac{kK_{voc}C_{voc}K_{ox}^{1/2}C_{ox}^{1/2}}{\left(1+K_{voc}C_{voc}+K_{water-voc}C_{water})(1+K_{ox}^{1/2}C_{ox}^{1/2}+K_{water-ox}C_{water}\right)}$												
	E_a_	k_o_	−ΔH_ox_	k_o.ox_	ΔH_voc_	k_o.voc_	−ΔH_water-ox_	k_o.water-ox_	−ΔH_water-red_	k_o.water-red_	RSS	R^2^
Pd/La-Ce-Zr-Al	143.8	3.03 × 10^12^	146.7	2.31 × 10^3^	74.5	7.68 × 10^−6^	86.2	8.03 × 10^−1^	88.4	6.92 × 10^−7^	6.1	0.985

E_a_, kJ/mol; ΔH_(ox,voc,water-ox,water-red)_, kJ/mol; k_o(ox,voc,water-ox,water-red)_, atm^−^^1^; k = k_o(ox,voc,water-ox,water-red)_.exp(−E_a_/RT); K_(ox,voc,water-ox,water-red)_ = k_o(ox,voc,water-ox,water-red)_.exp(ΔH_(ox,voc,water-ox,water-red)_/RT); −ΔH_(ox,voc,water-ox,water-red)_ = E_des_ − E_ads_; RSS—squared sum of residuals; R^2^—squared correlation coefficient.

**Table 7:** Reaction rate expressions and kinetics parameters for the applied MVK model.

Model: MVK-SDP, (Water Adsorbs on Oxidized and Reduced Sites, Slow Desorption of Products) $r=\frac{k_{red}k_{ox}C_{voc}C_{ox}}{\gamma k_{red}C_{voc}\left(1+K_{water-voc}.C_{water-voc}\right)+k_{ox}C_{ox}\left(1+K_{water-ox}.C_{water-ox}\right)+(k_{red}k_{ox}/k_{des})C_{voc}C_{ox}}$, γ=2												
	E_a_	k_o_	−ΔH_ox_	k_o.ox_	−ΔH_voc_	k_o.voc_	−ΔH_water-ox_	k_o.water-ox_	−ΔH_water-red_	k_o.water-red_	RSS	R^2^
Pd/La-Ce-Zr-Al	120.3	2.43 × 10^11^	62.4	6.99 × 10^6^	97.3	1.5 × 10^−7^	67.4	1.00 × 10^−6^	134.8	5.06 × 10^11^	5.0	0.995

E_a_, kJ/mol; ΔH_(ox,voc,water-ox,water-red)_, kJ/mol; k_o(ox,voc,water-ox,water-red)_, atm^−^^1^; k_(ox,voc,water-ox,water-red)_ = k_o(ox,voc,water-ox,water-red)_.exp(−E_a_/RT); K_(ox,voc,water-ox,water-red)_ = k_o(ox,voc,water-ox,water-red)_.exp(ΔH_(ox,voc,water-ox,water-red)_/RT); −ΔH_(ox,voc,water-ox,water-red)_ = E_des_ − E_ads_; RSS—squared sum of residuals; R^2^—squared correlation coefficient.

## Data Availability

The data presented in this study are available upon request from the corresponding author.
